# Change in Activities of Daily Living, Functional Capacity, and Life Satisfaction in Japanese Patients with Subacute Myelo-Optico-Neuropathy

**DOI:** 10.2188/jea.JE20090184

**Published:** 2010-11-05

**Authors:** Tetsuya Kamei, Shuji Hashimoto, Miyuki Kawado, Rumi Seko, Takatoshi Ujihira, Masaaki Konagaya

**Affiliations:** 1 Faculty of Medical Management and Information Science, Fujita Health University School of Health Sciences, Toyoake, Aichi, Japan; 2 Department of Hygiene, Fujita Health University School of Medicine, Toyoake, Aichi, Japan; 3 Faculty of Nursing, Fujita Health University School of Health Sciences, Toyoake, Aichi, Japan; 4 Health and Welfare Bureau, Nara Prefecture, Nara, Japan; 5 Department of Neurology, Suzuka National Hospital, Suzuka, Mie, Japan

**Keywords:** subacute myelo-optico-neuropathy, SMON, activities of daily living, functional capacity, life satisfaction, follow-up

## Abstract

**Background:**

There have been few reports on longitudinal change in activities of daily living (ADL), functional capacity, and life satisfaction in patients with subacute myelo-optico-neuropathy (SMON).

**Methods:**

A total of 1309 SMON patients 40 to 79 years of age underwent a medical examination conducted by the SMON Research Committee during the period from 1993 through 1995 (baseline) in Japan; 666 (51%) were followed-up after 12 years and were thus eligible for analysis. We calculated scores for ADL, functional capacity, and life satisfaction at baseline, and at 3, 6, 9, and 12 years after baseline, using data from medical examinations conducted in 1993 through 2007. The Barthel Index, the Tokyo Metropolitan Institute of Gerontology Index of Competence, and the patient’s response to the question “Are you satisfied with life?” were used to assess ADL, functional capacity, and life satisfaction, respectively.

**Results:**

As compared with baseline, the mean scores for ADL, functional capacity, and life satisfaction were all significantly lower after 12 years in men and women, with the exception of life satisfaction in women. The change in scores for functional capacity from baseline to year 12 was significantly associated with change in life satisfaction; however, the changes in ADL and age at baseline were not.

**Conclusions:**

We observed decreases in ADL, functional capacity, and life satisfaction among SMON patients. Our results suggest that a decrease in life satisfaction can be prevented by maintaining or improving functional capacity.

## INTRODUCTION

Subacute myelo-optico-neuropathy (SMON) is a disease caused by clioquinol intoxication and is characterized by subacute onset of sensory and motor disturbance in the lower extremities and visual impairment following abdominal symptoms.^1^^,^^2^ In Japan, the incidence of SMON peaked in the decade from 1960 to 1970.^3^^,^^4^ Currently, there are more than 2000 SMON patients, and they have a number of serious neurological symptoms.^4^

Some studies have reported that activities of daily living (ADL) are limited in SMON patients due to their neurological symptoms.^4^^–^^6^ In addition to ADL, functional capacity and life satisfaction are important in the lives of older SMON patients. Functional capacity includes instrumental self-maintenance, intellectual activities, and social role.^6^^,^^7^ In our previous report, we used cross-sectional data from medical examinations of SMON patients conducted by the SMON Research Committee to analyze the distribution and associations of ADL, functional capacity, and life satisfaction.^8^ However, there have been few studies on longitudinal change in ADL, and changes in functional capacity and life satisfaction have not been reported.^5^

In the present study, we examine longitudinal change and associations of ADL, functional capacity, and life satisfaction in SMON patients, using follow-up data collected from 1993 through 2007.

## METHODS

### Medical examination of SMON patients

SMON patients throughout Japan are offered annual examinations conducted by the SMON Research Committee with the support of the Japanese Ministry of Health, Labour and Welfare.^5^^,^^9^ In some prefectures, all resident SMON patients are examined within a 3-year interval. Information on neurological symptoms, ADL, functional capacity, and life satisfaction is collected by means of an interview and examination using a standardized survey form.

### Subjects

We analyzed data from medical examinations conducted during the period from 1993 through 2007. The data from 1993 through 1995 were used to determine the baseline status of the patients; data collected later were used for follow-up.

SMON patients who underwent a medical examination at 40 to 79 years of age during the period from 1993 through 1995 (baseline) were eligible for inclusion. Of 1410 patients, we excluded 34 who did not consent to the use of their medical examination data for analysis, and 67 with missing data on ADL, functional capacity, or life satisfaction at baseline. The data at baseline from 1309 patients (classified as patients for follow-up) were thus available for analysis. At 12 years after baseline, data from 666 patients (classified as patients eligible for analysis) were obtained from medical examinations conducted from 2005 through 2007. Table 1 shows the number of patients eligible for analysis by sex and age. Of 338 men and 971 women, 171 (51%) and 495 (51%), respectively, were eligible for analysis.

**Table 1.** Mean and standard deviation of scores for activities of daily living, functional capacity, and life satisfaction at baseline, by sex and age

Age at baseline (years)	Men				Women			
	
	No.	Activities of daily living	Functional capacity	Life satisfaction	No.	Activities of daily living	Functional capacity	Life satisfaction
40–49	8	100.0 ± 0.0	11.8 ± 1.3	3.0 ± 1.4	35	90.3 ± 14.2	10.7 ± 3.9	3.5 ± 1.2
50–59	45	95.6 ± 11.2	11.4 ± 2.5	3.3 ± 1.2	117	91.0 ± 12.3	11.1 ± 2.7	3.3 ± 1.3
60–69	88	91.9 ± 12.9	9.9 ± 3.4	3.4 ± 1.1	228	90.5 ± 13.0	9.9 ± 3.0	3.4 ± 1.1
70–79	30	89.8 ± 21.6	10.1 ± 3.6	3.6 ± 1.0	115	88.3 ± 15.8	9.0 ± 3.5	3.6 ± 1.1

Total	171	92.9 ± 14.3	10.4 ± 3.2	3.4 ± 1.1	495	90.1 ± 13.6	10.0 ± 3.2	3.4 ± 1.2

### ADL, functional capacity, and life satisfaction

The Barthel Index was used to measure ADL. Scores range from 0 to 100, and a higher score indicates higher ADL.^10^ The Tokyo Metropolitan Institute of Gerontology Index of Competence (TMIG Index) was used to measure functional capacity and ranges from 0 to 13, and a higher score indicates higher capacity.^11^^,^^12^ Life satisfaction was evaluated using the response to the question “Are you satisfied with life?” ^8^ The responses were grouped into 5 categories: satisfied, slightly satisfied, neutral, slightly dissatisfied, and dissatisfied. We assigned scores of 5, 4, 3, 2, and 1, respectively, to these categories.

### Statistical analyses

We calculated scores for ADL, functional capacity, and life satisfaction at baseline for patients eligible for analysis, using the data from medical examinations conducted from 1993 through 1995. Scores at 3, 6, 9, and 12 years after baseline were calculated using data from 1996 through 1998, 1999 through 2001, 2002 through 2004, and 2005 through 2007, respectively. Changes in mean scores from baseline values to those at 3, 6, 9, and 12 years after baseline were calculated, and 95% confidence intervals were estimated. Differences in mean scores at baseline and year 12 were tested using the paired *t* test. The associations between changes in scores for ADL, functional capacity, and life satisfaction from baseline to year 12 were examined using a linear regression model, with change in life satisfaction as a dependent variable and age at baseline and change in ADL and functional capacity as independent variables. The *t* test was used to examine the characteristics of patients eligible for analysis: their mean age and scores for ADL, functional capacity, and life satisfaction at baseline were compared with those of patients who were ineligible because data at 12 years were not obtained. Statistical analyses were conducted using SAS software, version 9.1 (SAS Institute, Inc., Cary, NC, USA).

### Ethical review

This study was approved in December 2005 by the Ethical Review Board for Epidemiological and Clinical Studies of the Fujita Health University School of Medicine.

## RESULTS

Table 1 shows the mean scores (± standard deviation) for ADL, functional capacity, and life satisfaction at baseline by sex and age among patients eligible for analysis. The mean ADL score was 92.9 in men and 90.1 in women, and the mean decreased with age, except in women 40 to 49 years of age. The mean score for functional capacity was 10.4 in men and 10.0 in women, and the mean decreased with age, except in men 70 to 79 years of age and women 40 to 49 years of age. The mean score for life satisfaction was 3.4 in men and 3.4 in women, and the mean increased with age, except in women 40 to 49 years of age.

**Figure 1.**
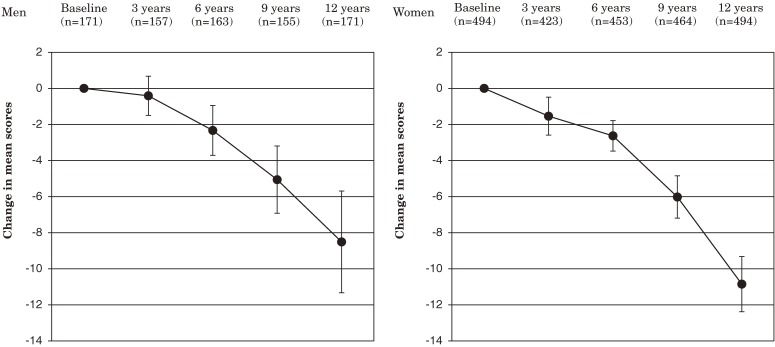
Change in mean scores for activities of daily living from baseline through year 12. Error bars indicate 95% confidence intervals.

**Figure 2.**
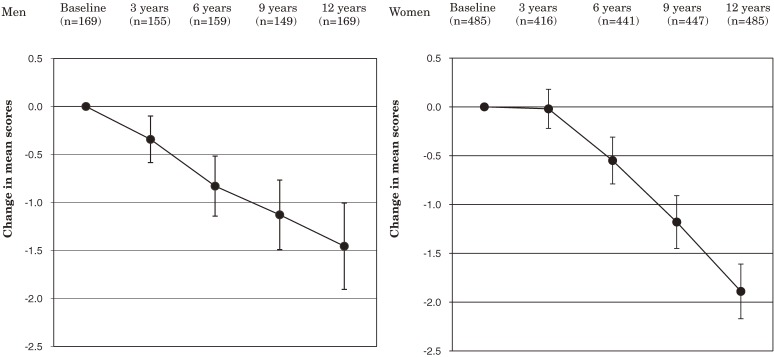
Change in mean scores for functional capacity from baseline through year 12. Error bars indicate 95% confidence intervals.

**Figure 3.**
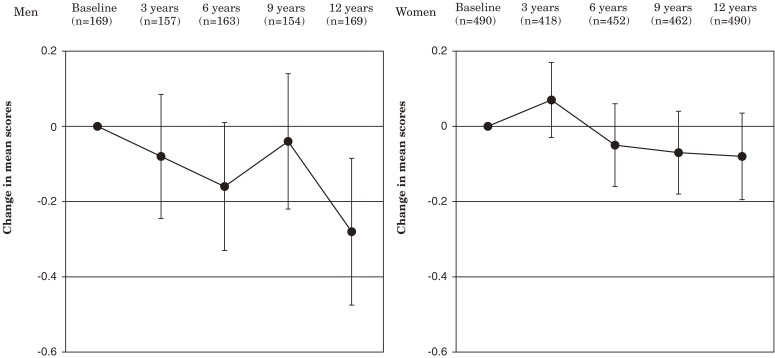
Change in mean scores for life satisfaction from baseline through year 12. Error bars indicate 95% confidence intervals.

Figures 1, 2, and 3 show the changes in the mean scores for ADL, functional capacity, and life satisfaction, respectively, from baseline through year 12 in patients eligible for analysis. At year 12, the mean score for ADL was significantly lower than baseline in men and women: −8.5 in men and −10.9 in women. The mean score for functional capacity was also significantly lower at year 12 in men and women: −1.5 in men and −1.9 in women. The mean score for life satisfaction was significantly lower at year 12 in men but not in women; the changes in the scores were −0.3 in men and −0.1 in women.

Table 2 shows the regression coefficients for the change in scores for life satisfaction at baseline and year 12. The change in scores for functional capacity from baseline to year 12 was significantly associated with change in life satisfaction, but the changes in ADL and age at baseline were not. The coefficient of determination was 8.2% in men and 1.7% in women.

**Table 2.** Regression coefficients for change in scores for life satisfaction at baseline and year 12

Independent variables	Men		Women	
	
	Regression coefficient^a^	*P* value^a^	Regression coefficient^a^	*P* value^a^
Intercept	−0.901		−0.844	
Age at baseline (years)	0.013	0.310	0.014	0.077
Change in activities of daily living^b^	0.005	0.487	−0.001	0.869
Change in functional capacity^b^	0.101	0.018	0.054	0.013

The coefficient of determination was 8.2% for men and 1.7% for women.

^a^Regression coefficient and *P* value from the linear regression model, with change in score for life satisfaction at baseline and year 12.

^b^Change in score for activities of daily living and functional capacity at baseline and year 12.

Table 3 shows the means (± standard deviation) for age and scores for ADL, functional capacity, and life satisfaction at baseline in patients eligible and ineligible for analysis. Mean age was significantly lower in eligible patients than in ineligible patients. The mean scores for ADL, functional capacity, and life satisfaction were significantly higher in eligible patients than in ineligible patients, except for life satisfaction in men.

**Table 3.** Mean and standard deviation of age and scores for activities of daily living, functional capacity, and life satisfaction at baseline in subjects eligible and ineligible for analysis

	Men			Women		
		
	Eligible subjects (*n* = 171)	Ineligible subjects (*n* = 167)	*P* value^a^	Eligible subjects (*n* = 495)	Ineligible subjects (*n* = 476)	*P* value^a^
Age	62.7 ± 7.9	67.6 ± 8.3	<0.001	63.3 ± 8.1	68.4 ± 8.3	<0.001
Activities of daily living	92.9 ± 14.3	87.0 ± 22.3	0.004	90.1 ± 13.6	86.9 ± 18.8	0.003
Functional capacity	10.4 ± 3.2	9.2 ± 3.9	0.002	10.1 ± 3.2	8.6 ± 3.8	<0.001
Life satisfaction	3.4 ± 1.1	3.3 ± 1.2	0.363	3.4 ± 1.2	3.3 ± 1.2	0.011

Subjects with follow-up data at 12 years after baseline were classified as eligible for analysis; all others were ineligible.

^a^*P* values were calculated using the *t* test.

## DISCUSSION

In an analysis of longitudinal data, we observed a significant decrease in ADL score among SMON patients. The decrease in mean score from baseline through year 12 was −8.5 in men and −10.9 in women. In a previous cross-sectional study of a random sample of the general population in Japan, the mean score on the Barthel Index for persons 50 to 59, 60 to 69, 70 to 79, and 80 to 94 years of age was between 96.9 and 99.9 in men and 93.2 and 99.2 in women.^13^ In a previous longitudinal study of a random sample of the older general population in Japan (NIPPON DATA80), the cumulative 5-year incidences of low ADL for adults 65 to 69 and 70 to 74 years of age were estimated at 4.3% and 4.8% in men and 4.7% and 9.2% in women, respectively.^14^ Assuming that a person with a Barthel Index score of 85 or higher is independent and that one with a score of 80 or lower has low ADL,^15^ the cumulative incidence of low ADL during the 12-year period of this study was estimated at 19.6% for 153 independent men with a mean age of 62 years and at 30.3% for 412 independent women with a mean age of 63 years. Although our results are not strictly comparable with those of the previous studies mentioned above, SMON patients may well experience a large decrease in ADL beyond the age-related decrease seen in the general population, due to their severe neurological symptoms.^5^^,^^13^^–^^17^

A longitudinal decrease was observed in the functional capacity and life satisfaction of SMON patients. The change in functional capacity from baseline through year 12 was significantly associated with change in life satisfaction when adjusted for age at baseline and change in ADL. Functional capacity, including instrumental self-maintenance and intellectual activities, plays an essential role in the social lives of adults.^6^^,^^12^^,^^18^ Our results suggest that measures for preventing a decrease in functional capacity and life satisfaction become gradually more important over time, and that a decrease in life satisfaction might be prevented by maintaining or improving the level of functional capacity.^8^^,^^19^ Further studies will be important for evaluating the causal relation between functional capacity as cause and decrease in life satisfaction as effect.

There are several limitations in the present study. The proportion of patients followed for 12 years after baseline was 51%. We obtained follow-up information from data from medical examinations carried out by the SMON Research Committee.^5^ Possible reasons for the losses to follow-up include death and worsening of the patient’s condition.^9^ Indeed, we observed that the mean scores for ADL, functional capacity, and life satisfaction at baseline were higher for patients who completed follow-up than for those who did not. Therefore, decreases in ADL, functional capacity, and life satisfaction might be larger in the overall SMON population than in our patients who completed follow-up.

The Barthel Index and TMIG Index used in this study are common tools for measuring ADL and functional capacity, respectively.^10^^–^^12^ We posed the question “Are you satisfied with life?” to measure life satisfaction. Although other indices for life satisfaction have been proposed,^20^ questions similar to ours were used in several previous studies.^21^^,^^22^ We do not know the reliability and validity of such questions, however. The results obtained from such questions thus require careful interpretation.

In regression analysis, the coefficient of determination with change of life satisfaction as a dependent variable was low. Previous studies indicate that social and economic factors were strongly associated with life satisfaction in older populations.^23^^,^^24^ It would be important to consider such factors in an analysis of associations between ADL, functional capacity, and life satisfaction in SMON patients.

In conclusion, ADL, functional capacity, and life satisfaction decreased in SMON patients. Our results suggest that the decrease in life satisfaction might be prevented by maintaining or improving the level of functional capacity.
